# Holidays for Slum Children

**Published:** 1910-06-25

**Authors:** R. Melvill Beachcroft

**Affiliations:** Chairman.


### INSTITUTIONAL LETTER-BOX.

### HOLIDAYS FOR SLUM CHILDREN.

*To the Editor of* THE HOSPITAL.

SIR,—The holiday season has already commenced, and most of us will, as a matter of course, in due time be getting away to the sea or to the moors. There are many, however, to whom a fortnight's holiday is a matter of chance and good luck: among whom are thousands of the poor children of London; and it is on their behalf that I plead for your readers' kindly thought and generosity. The small sum of 10s. will give a London slum-child a fortnight's holiday in a clean, comfortable, happy home by the sea or in the heart of the country. There must be thousands of people who love to give pleasure to little children, and to whom the expenditure of a mere half-sovereign is a small affair. But it is surprising that more do not avail themselves of this means of giving a thoroughly beneficial present to those who are in real need, and truly deserve the gift. Will you therefore allow me to beg your readers who feel disposed to invest a sum in this way—though the amount need not be restricted to 10s.—to send me their contributions for the work of the Holiday Homes' Fund of the Ragged School Union? Every half-sovereign received will give a fortnight's sheer delight to some little lad or lass who otherwise would not experience the vast difference between the playground of the London streets and the seashore and green fields.

I am, Sir, your obedient servant,
R. MELVILL BEACHCROFT, Chairman.

